# Associations between sleep disorders, anxiety, depression, and the phases of sarcopenia to severe sarcopenia: findings from the WCHAT study

**DOI:** 10.3389/fpubh.2025.1539729

**Published:** 2025-08-28

**Authors:** Zhigang Xu, Ya Ma, Huang Ning, Shuli Jia, Gongchang Zhang, Xin Xia, Fengjuan Hu, Meiling Ge, Xiaolei Liu, Birong Dong

**Affiliations:** ^1^The Affiliated Traditional Chinese Medicine Hospital, Southwest Medical University, Luzhou, China; ^2^National Clinical Research Center of Geriatrics, West China Hospital, Sichuan University, Chengdu, China

**Keywords:** sarcopenia, Western China, multi-ethnic, sleep quality, anxiety and depression

## Abstract

**Background:**

Sarcopenia not only leads to impaired physical function but also may be associated with changes in sleep and mental health as individuals age. Research on the relationships between sleep disorders, anxiety, and depression and adult-onset sarcopenia is limited, however, with no reports of the associations between them and different severity of sarcopenia. The objective of this research endeavor is to investigate the associations between sarcopenia and sleep disturbances, anxiety, as well as depression, within a multi-ethnic population in western China.

**Method:**

We conducted a cross-sectional study consisting of 4,500 participants from the WCHAT study. The diagnostic method recommended by the Asian Working Group for Sarcopenia in 2019 was used to screen for sarcopenia. The Pittsburgh Sleep Quality Index (PSQI), the 7-item Generalized Anxiety Disorder Questionnaire (GAD-7), and the 15-item Geriatric Depression Scale (GDS-15) were used to assess sleep quality, anxiety, and depression, respectively. The relationships among sleep, anxiety, depression, and the different sarcopenia subgroups were evaluated by using multivariate regression models. In addition, subgroup of gender analysis were performed.

**Results:**

Among the 4,500 participants surveyed in the western region of China, 408 (9.06%) were diagnosed with sarcopenia and 618 (13.73%) with severe sarcopenia. A total of 2,515 individuals (55.88%) had poor sleep quality, while 842 (18.71%) suffered from anxiety, and 1,045 (23.22%) had depression. Good sleeping quality were negatively associated with severe sarcopenia (OR: 0.80, 95%CI 0.66–0.97) in model 1, whereas depression was positively associated with severe sarcopenia in three models (model 1: OR: 1.39, 95%CI 1.13–1.71; model 2: OR: 1.46, 95%CI 1.16–1.85; model 3: OR: 1.43, 95%CI 1.13–1.81). However, anxiety status was not associated with sarcopenia in our study.

**Conclusion:**

It was found that good sleep quality were negatively associated with severe sarcopenia, and depression was positively associated with severe sarcopenia. These findings suggested that early intervention in sleep quality and depression may be one of the effective strategies to delay or reduce the severity of sarcopenia.

**Clinical trial registration:**

https://www.chictr.org.cn/, identifier ChiCTR1800018895.

## Background

The aging of China’s population is accelerating. It is expected that by 2050, China’s population over 65 years old will reach 400 million, including 150 million people over 80 years old, and the public medical burden will continue to increase (1). Therefore, age-related diseases have attracted increasing attention. After middle age, the functions of the human body gradually decline (2). Certain groups of people, especially older adults, may experience an increased incidence of sleep disorders, anxiety, and even depression (3–5). These psychological changes may also influence hormone levels, affecting protein synthesis and thus the maintenance of muscle mass (6). In addition, sleep disorders, anxiety, and depression may in turn induce changes in daily life and diet that may lead to changes in muscle metabolism (7).

Sarcopenia is a progressive systemic disease of the skeletal muscles that occurs with aging and is associated with various adverse outcomes (8), including an increased likelihood of hospitalization and even death. Older adults with severe muscle loss have an increased risk of short-term mortality, making sarcopenia one of the predictors of mortality in community-dwelling older adults (9). This also indicates that early intervention in the prevention and treatment of sarcopenia is particularly important in healthcare services in China.

Research indicates that poor sleep quality is associated with physical function, mortality, frailty, and falls in older adults (9–11). Sleep may impact muscle mass and strength through metabolism, hormones, and immune factors, which in turn may affect physical performance (12). Anxiety is common in the older population and is often comorbid with depression and associated with cognitive decline (13). Cognitive function has been significantly correlated with sarcopenia (14). In ethnically diverse regions such as China, there is limited research on the associations between sleep, anxiety, depression, and sarcopenia.

Due to differences in ethnicity, lifestyle, dietary habits, economies, geography, and beliefs between Western and Asian countries, separate sarcopenia working groups have been formed in Europe and Asia. These groups have formulated assessment methods and diagnostic criteria for sarcopenia, each with its own variations. This has led to disparities in the reported prevalence of regional sarcopenia among different countries. China is a populous Asian country with a multi-ethnic population. This suggests the value of investigating the incidence and characteristics of sarcopenia in multi-ethnic regions, such as those of western China. According to the recommendations of the Asian Working Group on Sarcopenia, sarcopenia as diagnosed in medical institutions or clinical research can be further divided into diagnosed sarcopenia and severe sarcopenia (15). Besides, most studies have focused only on correlations between a single factor associated with sleep or depression and overall sarcopenia, and there is no information on the relationship between anxiety and sleep, depression, and different sarcopenia subgroups, nor are there definitive research conclusions. Sleep disturbances (e.g., insomnia, sleep apnea) and mental health disorders (anxiety, depression) are highly comorbid with sarcopenia, yet their bidirectional relationships are underexplored. Chronic sleep deprivation disrupts muscle protein synthesis and exacerbates inflammation, while anxiety and depression correlate with reduced physical activity and poor nutritional intake—key modifiable risk factors for sarcopenia.

This study was based on data from the Western China Health and Aging Trends (WCHAT) longitudinal multi-center cohort study (16). This study is a cross-sectional study investigated the associations between sleep quality, anxiety, depression, and sarcopenia severity using a multi-ethnic population-based sample. Its findings could inform multidisciplinary interventions targeting sleep hygiene and mental health to mitigate sarcopenia progression, particularly in low-resource settings where sarcopenia is often underdiagnosed. Given the aging global population, elucidating these pathways is critical for reducing healthcare burdens and improving quality of life in older adults.

## Methods

### Study design and participants

The study population consisted of individuals from multiple provinces and cities in the western region of China, representing various ethnic groups. This study relied on the ongoing prospective cohort study WCHAT, the methodology and study design of which has been previously published (16). The Ethics Committee of West China Hospital, Sichuan University, China reviewed and approved this study (reference number: 2017–445), and all participants signed the informed consent form (16). The participants were recruited from four provinces in western China, namely, Sichuan, Xinjiang, Guizhou, and Yunnan, with a focus on Sichuan. The participants represent various ethnic groups, including Han, Tibetan, Qiang, Yi, Hui, Zhuang, and Miao.

Inclusion criteria for participants: living in the region for at least 3 years; age ≥50 years; voluntary participation in the study. Exclusion criteria: expected lifespan of less than 6 months; acute diseases of important organs, such as the heart, liver, and kidneys, and severe diseases such as respiratory failure; refusal to participate in the survey.

A total of 7,536 participants were recruited in the multi-ethnic regions in western China. Bioelectrical impedance analysis (BIA) data were obtained from 4,500 individuals, as well as information on sleep, anxiety, and depression scales, which were ultimately used to analyze individuals with sarcopenia. All data collection personnel involved in this study received rigorous training, and health checks were conducted by relevant professional technicians. This study was approved by relevant committees in china, and every patients provided written consent. All methods used followed relevant regulations.

## Measures

### Sarcopenia screening

Sarcopenia is characterized by an accelerated loss of muscle mass and function. For primary health care or community-based health purposes, the Asian Working Group on Sarcopenia (AWGS) defined “possible sarcopenia” as low muscle strength or physical function (15). In this study, we followed the screening methods recommended by the AWGS, using the sarcopenia assessment pathways corresponding to clinical research, categorizing sarcopenia into diagnosed and severe sarcopenia. The primary diagnostic criterion for individuals with diagnosed sarcopenia was a decrease in muscle mass. These individuals also met one of two secondary criteria, namely, a decline in handgrip strength or gait speed. Individuals suffering from severe sarcopenia met both of these secondary criteria. The specific assessment methods and data collection are described below.

Muscle mass was assessed by bioimpedance analysis (BIA) using the INbody770 body composition instrument for data collection. The reliability of this instrument was validated in the relevant Chinese population (17, 18). Following the AWGS2019 recommendations, we used a cutoff value of 7.0 kg/m^2^ for men and 5.7 kg/m^2^ for women for the determination of the skeletal muscle mass index (ASMI) (15).

Handgrip testing is used as a reflection of muscle strength. The handgrip strength of the subjects’ dominant hand was measured using a dynamometer (EH101; Camry, Zhongshan). During the measurement, the subjects were asked to stand with their feet naturally apart, arms hanging down, and to perform the maximum grip strength test on two separate occasions, recording the maximum value. The standard for weak grip strength is <18 kg for females and <28 kg for males (15).

The general gait speed test requires participants to wear flat shoes and may use a walking aid for measuring walking speed. Participants can rest during the measurement but should not sit down. The AWGS recommends a critical value of 1.0 m/s for gait speed in individuals with muscle weakness.

In our study, following the AWGS2019 criteria, sarcopenia was defined as low muscle mass and low handgrip strength or low gait speed. And severe sarcopenia was defined as low muscle mass combined with low handgrip strength and low gait speed.

### Sleep quality assessment

As an indicator of subjective sleep quality in the past month, the Pittsburgh Sleep Quality Index (PSQI) was used to gauge sleep quality. It consists of 19 items and is commonly used in the diagnosis of sleep disorders in both clinical and research settings, serving as a standardized assessment for patients with sleep difficulties. A PSQI score above 5 indicates poor sleep quality, while a PSQI score below 5 indicates good sleep quality.

### Anxiety assessment

The Generalized Anxiety Disorder Questionnaire (GAD-7) was used to measure anxiety. Currently, GAD-7 is one of the most widely used measures for anxiety assessment in clinical practice and research due to its diagnostic reliability and high efficiency (19).

### Depression assessment

To assess depressive symptoms, we used the 15-item Geriatric Depression Scale, which was developed to evaluate the unique symptoms commonly exhibited by depressed older adults, such as somatic symptoms, anxiety, and cognitive decline. A GDS-15 score above 5 indicates depressed mood (20).

### General information on the study population

This included demographics (sex, age, ethnicity, marital status, employment status, living arrangements), lifestyle factors (alcohol consumption, smoking status), and health conditions (chronic diseases, such as hypertension, diabetes, heart disease, and COPD).

### Statistical analysis

Data were analyzed using SPSS 22.0. Continuous variables are expressed as mean and standard deviation (X ± SD) and were compared using *t*-tests. Categorical variables are presented as percentages and were analyzed with *χ*^2^ tests. Descriptive statistics were used to describe demographic and clinical characteristics, with Chi-square and Kruskal-Wallis tests for analysis. Multivariate logistic regression analyses were performed to evaluate the relationship between sarcopenia with sleep quality, depression and anxiety status with corresponding 95% confidence interval (CI). We contrasted three models as follows: model 1, adjusted for age, gender and ethnics; model 2: adjusted for age, gender, ethnics, marriage status, living alone, life styles (smoking, drinking tea); model 3: adjusted for age, gender, ethnics, marriage status, living alone, life styles (smoking, drinking tea), educational level, and chronic diseases.

## Results

**Figure 1:**
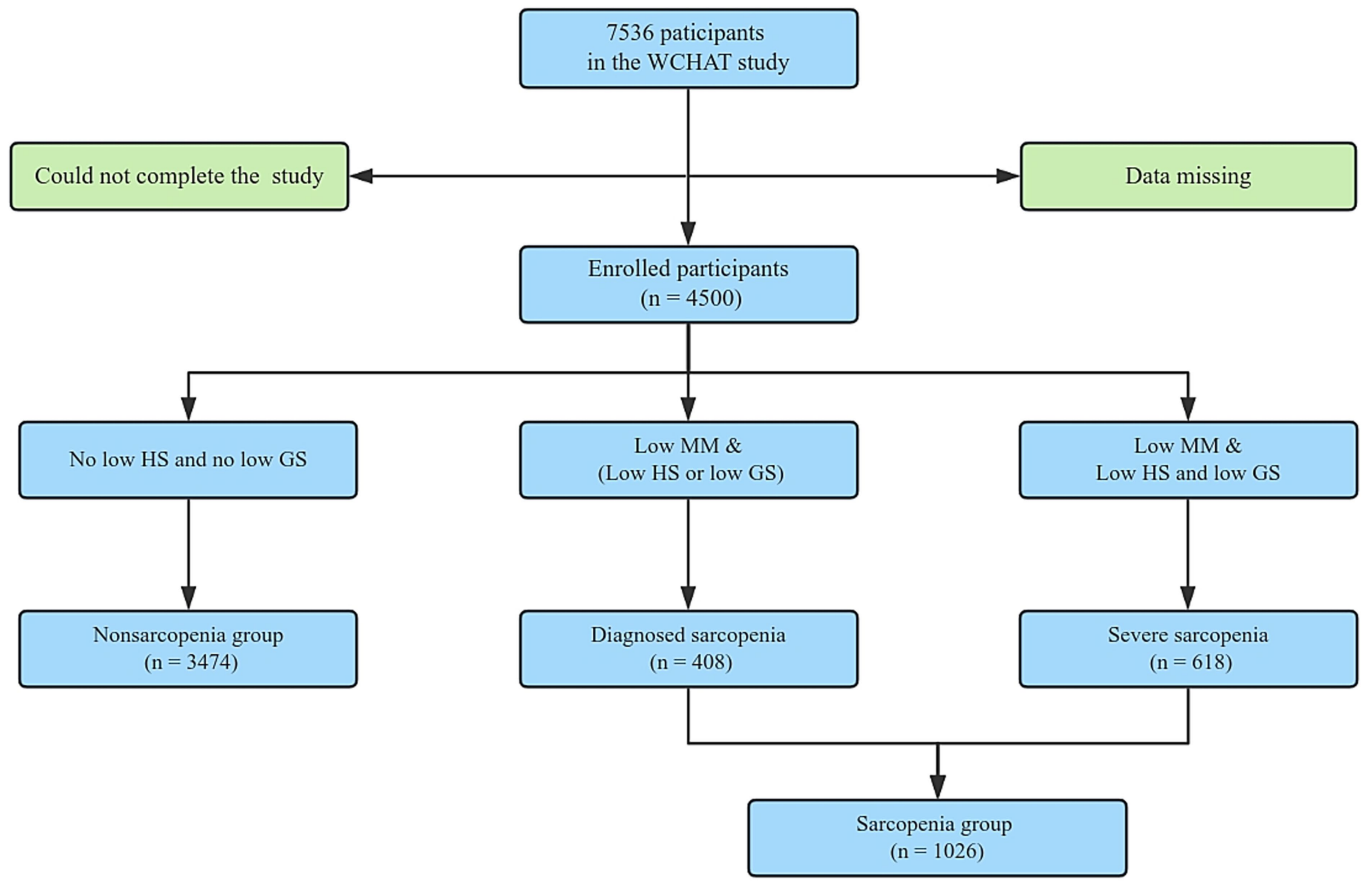
Flowchart of the study. Participants were recruited from the multi-ethnic regions in western China, following the diagnostic procedure of AWGS2019 for sarcopenia. GS, Gait speed; HS, Handgrip strength; MM, Muscle mass.

This study recruited 7,536 participants (age>50 years) in multi-ethnic communities in western China. However, due to the failure of some community participants to complete relevant examinations and partial data loss, 4,500 participants were finally enrolled. Figure 1 shows the sarcopenia screening process for participants, which is based on the sarcopenia screening process recommended by AWGS 2019 for medical institutions and clinical research.

Among the overall cohort, there were 3,474 cases (77.2%) in the non-sarcopenia group, 408 cases (9.07%) in the diagnosed sarcopenia group, 618 cases (13.73%) in the severe sarcopenia group, and a total of 1,026 cases (22.8%) in the sarcopenia group. Table 1 shows the demographic and clinical characteristics of participants in multi-ethnic areas in the western region of China. Significant differences were observed in terms of ethnic group, age, gender, education level, smoking history, ASMI, chronic diseases, sleeping quality, living alone and depression, while no significant differences were seen in terms of drinking history, anxiety status, marital status and chronic disease.

**Table 1:** General demographic and clinical characteristics of individuals with different degrees of sarcopenia (*n* = 4,500).

Characteristics	Non-sarcopenia	Diagnosed sarcopenia	Severe sarcopenia	*p*-value
	(*n* = 3,474)	(*n* = 408)	(*n* = 618)	
Age (Years)	60.99 ± 7.55	63.15 ± 7.66	69.63 ± 8.57	*p* < 0.001
Different age stratification				*p* < 0.001
50 ≤ Age<65	1719 (49.5%)	156 (38.2%)	97 (15.7%)	
65 ≤ Age<74	1,342 (38.6%)	181 (44.4%)	217 (35.1%)	
75 ≤ Age<84	385 (11.1%)	62 (15.2%)	237 (38.3%)	
85 ≤ Age	28 (0.8%)	9 (2.2%)	67 (10.8%)	
Gender				*p* < 0.001
Male	1,186 (34.1%)	187 (45.8%)	254 (41.1%)	
Female	2,288 (65.9%)	221 (54.2%)	364 (58.9%)	
Smoking history				*p* < 0.001
Yes	506 (15.4%)	98 (25.1%)	134 (23.1%)	
No	2,780 (84.6%)	293 (74.9%)	445 (76.9%)	
Drinking alcohol				*p* = 0.334
Yes	822 (25.0%)	110 (28.1%)	139 (24.0)	
No	2,463 (75.0%)	282 (71.9%)	440 (76.0%)	
Ethnics				*p* < 0.001
Han	1,423 (41.0%)	205 (50.2%)	309 (50.0%)	
Zang	975 (28.1%)	107 (26.2%)	151 (24.4%)	
Qiang	898 (25.8%)	60 (14.7%)	92 (14.9%)	
Yi	131 (3.8%)	27 (6.6%)	55 (8.9%)	
Others	47 (1.4%)	9 (2.2%)	11 (1.8%)	
Education level				*p* < 0.001
No formal education	944 (27.2%)	116 (28.4%)	234 (37.9%)	
Elementary school	1,106 (31.8%)	131 (32.1%)	211 (34.1%)	
Middle school	753 (21.7%)	79 (19.4%)	89 (14.4%)	
High school and above	671 (19.3%)	82 (20.1%)	84 (13.6%)	
Marital status				*p* = 0.188
Singlehood	22 (0.7%)	2 (0.5%)	7 (1.2%)	
Married	2,837 (86%)	330 (84.2%)	426 (72.9%)	
Divorced	52 (1.6%)	2 (0.5%)	13 (2.2%)	
Widowed	388 (11.8%)	58 (14.8%)	138 (23.6%)	
Sleep quality				*p* = 0.008
PQSI≤5	1765 (53.6%)	214 (54.6%)	272 (46.8%)	
PQSI> 5	1,528 (46.4%)	178 (45.4%)	809 (53.2%)	
Living alone				*p* = 0.004
Yes	144 (4.1%)	22 (5.4%)	44 (7.1%)	
No	3,330 (95.9%)	386 (94.6%)	574 (92.9%)	
ASMI mean (±SD)	6.89 (0.84)	5.85 (0.67)	5.65 (0.72)	*p* < 0.001
Chronic diseases				*p* = 0.064
Yes	1,482 (45.0%)	159 (40.6%)	280 (48.2%)	
No	1811 (55.0%)	233 (59.4%)	381 (51.8%)	
Depressive status				*p* = 0.011
GDS-15, <5	2,687 (77.3%)	322 (78.9%)	446 (72.2%)	
GDS-15, ≥5	787 (22.7%)	86 (21.1%)	172 (27.8%)	
Anxiety status				*p* = 0.309
GAD-7 < 5	2,812 (80.9%)	343 (84.1%)	503 (81.4%)	
GAD-7 ≥ 5	662 (19.1%)	65 (15.9%)	115 (18.6%)	

Other ethnicities including Zhuang, Mongolian, Uygur, Bai, Dong, Manchu, Hui, and Tujia. Means ± standard deviation was shown. Data are shown using % or mean (standard deviation). *p* values were calculated with chi-squared tests and student’s *t* tests for categorical and continuous variables, respectively. ASMI, Appendicular Skeletal Muscle Index; GDS, Geriatric Depression Scale, and GAD, Generalized Anxiety Disorder.

Table 2 presents potential risk factors associated with different groups of sarcopenia. Specifically, compared to the highest age group, individuals with younger age group were less likely to develop into sarcopenia, especially the severe sarcopenia. Compared to the male group, female group was less likely to develop into sarcopenia (OR 0.62, 95%CI = 0.47–0.82). Additionally, individuals who did not have smoking history were found to be less likely to have severe sarcopenia (OR = 0.69, 95% CI = 0.53–0.91). And those individuals who were Qiang group were also less likely to develop into sarcopenia (OR = 0.34, 95% CI = 0.16–0.74) and severe sarcopenia (OR = 0.38, 95% CI = 0.18–0.77) compared to the others. Compared to the highest education level group, those with no formal education level was more likely to develop into severe sarcopenia (OR = 2.78, 95% CI = 1.97–3.91). Finally, compared to widowed status, the presence of marriage status was also less likely to develop into diagnosed sarcopenia (OR = 0.70, 95% CI = 0.51–0.97) and severe sarcopenia (OR = 0.43, 95% CI = 0.33–0.54).

**Table 2:** Multivariate regression analysis of risk factors associated with different sarcopenia groups in multi-ethnic areas in western China (*n* = 4,500).

Factors	Diagnosed sarcopenia (*n* = 408)				Severe sarcopenia (*n* = 618)			
	*β*	OR	95%CI	*p*-value	*β*	OR	95%CI	*p*-value
Age								
50 ≤ Age<65	−1.21	0.30	0.13–0.67	*p* = 0.003	−3.60	0.03	0.02–0.05	*p* < 0.001
65 ≤ Age<74	−0.81	0.45	0.20–0.99	*p* = 0.046	−2.56	0.08	0.05–0.13	*p* < 0.001
75 ≤ Age<84	−0.61	0.54	0.24–1.23	*p* = 0.142	−1.24	0.29	0.17–0.47	*p* < 0.001
85 ≤ Age^a^		1				1		
Gender								
Female	−0.47	0.62	0.47–0.82	*p* = 0.001	−0.54	0.58	0.46–0.74	*p* < 0.001
Male^a^		1				1		
Smoking history								
No	−0.31	0.74	0.54–1.00	*p* = 0.053	−0.37	0.69	0.53–0.91	*p* = 0.009
Yes^a^		1				1		
Drinking alcohol								
No	0.005	1.005	0.77–1.32	*p* = 0.969	0.17	1.19	0.94–1.51	*p* = 0.156
Yes^a^		1				1		
Ethnics groups								
Han	−0.24	0.78	0.37–1.65	*p* = 0.522	−0.12	0.88	0.44–1.76	*p* = 0.724
Zang	−0.55	0.58	0.27–1.23	*p* = 0.153	−0.57	0.57	0.28–1.15	*p* = 0.114
Qiang	−1.08	0.34	0.16–0.74	*p* = 0.007	−0.97	0.38	0.18–0.77	*p* = 0.008
Yi	−0.07	0.93	0.40–2.17	*p* = 0.866	0.14	1.15	0.54–2.47	*p* = 0.719
Others^a^		1				1		
Education level								
No formal education	0.14	1.15	0.82–1.62	*p* = 0.409	1.02	2.78	1.97–3.91	*p* < 0.001
Elementary school	−0.04	0.96	0.70–1.32	*p* = 0.803	0.67	1.96	1.41–2.74	*p* < 0.001
Middle school	−0.21	0.81	0.57–1.16	*p* = 0.252	0.19	1.22	0.84–1.76	*p* = 0.302
High school and above^a^		1				1		
Living alone								
No	−0.07	0.93	0.57–1.53	*p* = 0.776	−0.04	0.96	0.65–1.42	*p* = 0.963
Yes^a^		1				1		
Chronic diseases								
No	0.27	1.30	1.05–1.62	*p* = 0.017	−0.01	0.99	0.82–1.19	*p* = 0.891
Yes^a^		1				1		
Marital status								
Single hood	−0.64	0.53	0.12–2.37	*p* = 0.406	−0.10	0.90	0.36–2.24	*p* = 0.822
Married	−0.36	0.70	0.51–0.97	*p* = 0.031	−0.85	0.43	0.33–0.54	*p* < 0.001
Divorced	−1.27	0.28	0.07–1.18	*p* = 0.083	−0.13	0.88	0.46–1.70	*p* = 0.706
Widowed^a^		1				1		

OR odds ratio, CI confidence interval.

a The variable was the reference.

Table 3 showed the results of the multivariate logistic regression analysis of sleep quality, depression status and anxiety status with sarcopenia groups were presented in three models. In model 1 which only adjusted age, gender and ethnics groups, good sleeping quality was negatively associated with severe sarcopenia (OR = 0.80, 95% CI = 0.66–0.97). While depression status was positively associated with severe sarcopenia (OR = 1.39, 95% CI = 1.13–1.71). After adjusting for all the potential confounders in model 3, the sleeping quality was not significantly associated with severe sarcopenia, while the depression status was still positively associated with severe sarcopenia (OR = 1.43, 95% CI = 1.13–1.81). However, multivariate logistic regression analysis with adjustment for confounding factors in three models showed no significant association between anxiety and different types of sarcopenia.

**Table 3:** Multiple regression analysis of sleeping quality, depression status and anxiety status with sarcopenia status among multi-ethnics in the west China communities (*N* = 4,500).

Sarcopenia		Model 1, OR (95%CI), *p* value	Model 2, OR (95%CI), *p* value	Model 3, OR (95%CI), *p* value
Sleeping quality	PQSI> 5	1.0(Ref)	1.0(Ref)	1.0(Ref)
	PQSI≤5	0.98 (0.79–1.22), 0.879	1.02 (0.82–1.26), 0.882	0.99 (0.79–1.23), 0.895
Depression status	GDS-15, <5	1.0 (Ref)	1.0 (Ref)	1.0 (Ref)
	GDS-15, ≥5	0.99 (0.78–1.23),0.947	1.10 (0.83–1.46), 0.492	1.12 (0.84–1.47), 0.446
Anxiety status	GAD-7<5	1.0 (Ref)	1.0 (Ref)	1.0 (Ref)
	GAD-7 ≥ 5	0.94 (0.71–1.25),0.686	0.92 (0.69–1.23), 0.585	0.94 (0.70–1.25), 0.653
Severe sarcopenia				
Sleeping quality	PQSI> 5	1.0(Ref)	1.0 (Ref)	1.0 (Ref)
	PQSI≤5	0.80 (0.66–0.97), 0.022	0.83 (0.69–1.00), 0.056	0.83 (0.68–1.00), 0.055
Depression status	GDS-15, <5	1.0 (Ref)	1.0 (Ref)	1.0 (Ref)
	GDS-15, ≥5	1.39 (1.13–1.71),0.002	1.46 (1.16–1.85), 0.001	1.43 (1.13–1.81), 0.003
Anxiety status	GAD-7 < 5	1.0 (Ref)	1.0 (Ref)	1.0 (Ref)
	GAD-7 ≥ 5	1.09 (0.86–1.38),0.473	1.09 (0.86–1.39), 0.460	1.09 (0.86–1.39), 0.477

OR, odds ratio; CI, confidence interval; Model 1: adjusted for age, gender and ethnics. Model 2: adjusted for age, gender, ethnics, marriage status, living alone, life styles (smoking, drinking tea). Model 3: adjusted for age, gender, ethnics, marriage status, living alone, life styles (smoking, drinking tea), educational level, and chronic diseases.

Table 4 shows that after gender stratification, it showed a significant association between depression and severe sarcopenia in female group (OR = 1.46, 95%CI = 1.1–1.93), but not in male group in the multivariate logistic regression analysis with full adjustment for confounding factors. No significant association were found between sleeping quality, anxiety status with sarcopenia after gender stratification.

**Table 4:** Association of sleeping quality, depression and anxiety with different sarcopenia groups in the multi-ethnic population of western China after gender stratification (*n* = 4,500).

	Adjusted model					
	Sleeping quality		Anxiety		Depression	
	Adjusted^b^ OR (95%CI)	*p*-value	Adjusted^b^ OR (95%CI)	*p*-value	Adjusted^b^ OR (95%CI)	*p*-value
Male						
Diagnosed sarcopenia^a^	1.19 (0.84–1.68)	*p* = 0.325	1.05 (0.63–1.75)	*p* = 0.842	1.01 (0.64–1.59)	*p* = 0.965
Severe sarcopenia^a^	0.76 (0.56–1.03)	*p* = 0.073	1.22 (0.79–1.89)	*p* = 0.377	1.17 (0.78–1.75)	*p* = 0.454
Female						
Diagnosed sarcopenia^a^	0.85 (0.63–1.13)	*p* = 0.262	0.85 (0.60–1.20)	*p* = 0.350	1.1 (0.77–1.57)	*p* = 0.588
Severe sarcopenia^a^	0.90 (0.70–1.16)	*p* = 0.416	0.97 (0.74–1.29)	*p* = 0.854	1.46 (1.1–1.93)	*p* = 0.008

OR, Odds ratio; CI, Confidence interval.

a Non-sarcopenia was the reference.

b Adjusted for age, marriage status, smoking history, drinking alcohol, ethnics group, education level, living alone, chronic diseases by logistic regression.

## Discussion

This study analyzed sarcopenia in a multi-ethnic population in western China, using the diagnostic and classification methods recommended by the 2019 AWGS for sarcopenia. The clinical screening results divided the study participants into three groups, namely, the non-sarcopenia, diagnosed sarcopenia, and severe sarcopenia groups. The prevalence of sarcopenia varies considerably depending on the actual methods used in different studies and the cutoff values chosen, as shown by studies from both European and American countries. This variation is influenced by the method used for muscle mass assessment with different diagnostic instruments, as well as factors such as ethnicity, place of residence, and age (21, 22). The present study found a 22.8% prevalence of sarcopenia, in contrast to a figure of 14.4% reported by Taiwanese researchers for individuals over the age of 65 using the diagnostic criteria of the European Working Group on Sarcopenia in Older People (22). The prevalence of sarcopenia is lower in Taiwan than in other regions. Apart from differences in diagnostic criteria, the results of the present study may be related to the multi-ethnic nature of the population and lower living standards in the western regions. Although our study included a wide age range, we found that the incidence of sarcopenia varies among different ethnic groups and varies in terms of the different types of sarcopenia. These differences among different ethnic groups may be related to genetics, dietary habits, and even religious beliefs, all of which require further in-depth research.

### Sleeping quality and sarcopenia

The quality of sleep is an important factor in maintaining physical and mental health, and disruptions or changes in circadian rhythms are associated with the development of many chronic diseases, including sarcopenia. Sleep quality is a multidimensional structure that includes sleep latency, awakening after sleep onset, frequency and number of awakenings, as well as subjective reports of feelings and mental state upon waking. Sleep quality is associated with reduced quality of life, increased incidence of disease, and higher mortality rates among older adults (23, 24).

There is research indicating that the sleep–wake cycle is associated with the maintenance of skeletal muscle, playing a crucial role in many physiological activities, muscle structure, and the metabolism of skeletal muscle. Many hormones, such as insulin, glucagon, cortisol, and growth hormone, exhibit circadian oscillations. The activity of certain metabolic enzymes and transport systems involved in the metabolism of cholesterol, glucose, and lipid receptors is also regulated by the circadian system. Therefore, disruption of the sleep–wake cycle may affect skeletal muscle metabolism (25). Sleep disorders have been observed to increase stress hormone levels, such as cortisol, which may trigger muscle breakdown and inhibit muscle protein synthesis, leading to the occurrence or progression of sarcopenia (26). At the same time, sleep disorders may disrupt the balance of growth hormones, potentially leading to sarcopenia (27). Sleep deprivation is also related to inflammatory responses, resulting in exacerbated systemic inflammation (28). Systemic inflammation can accelerate muscle degeneration and hinder muscle function, potentially worsening sarcopenia (29). Research indicates that sleep disorders can also affect neurological function and motor control which may impair neuromuscular coordination and gradually lead to muscle damage. In disrupted circadian rhythm models, the absence of the Bma/1 gene has been observed to lead to sarcopenia and several pathological muscle diseases, including reduced mitochondrial density and altered mitochondrial respiration, fiber type displacement, and impaired muscle segment structure (30, 31). Chronic sleep deprivation may accelerate sarcopenia through disrupted growth hormone signaling and elevated pro-inflammatory cytokines (25). Research has shown an association between sleep initiation and/or maintenance and sarcopenia in older Japanese individuals (32). This study found a significant correlation between sleep quality and severe sarcopenia, but no significant correlation with diagnosed sarcopenia. Thus, in the aging population with sleep disorders, improving sleep quality may slow down muscle loss and prevent or delay the onset of severe sarcopenia.

### The relationship between anxiety, depression, and sarcopenia

Anxiety shows strong comorbidity with depression in older adults, and it is also associated with cognitive decline (13). Anxiety and depression thus form a common geriatric syndrome. Both are also related to circadian rhythms, which are closely linked to the function of skeletal muscles (30). Research has shown a close association between anxiety and depression and the fragmentation of the 24-h activity rhythm in individuals middle-aged and older (33). Anxiety, depression, and physical activity are significantly correlated, with lower levels of daily activity being a core feature of mood disorders (34). Currently, there is limited research on anxiety disorders, although some research findings indicate that they can influence depression in terms of psychomotor retardation, lower levels of daily activities, and circadian rhythm disturbances (35). There are many potential mechanisms linking sarcopenia with mental disorders (35). Brain-derived neurotrophic factor, a neurotrophic factor produced by skeletal muscle, is associated with anxiety disorders (36). Research has found that chronic inflammation plays a vital role in the progression of sarcopenia and affects the evolution of anxiety disorders (37–39). Additionally, there are many common lifestyle risk factors for both sarcopenia and anxiety disorders, including physical inactivity, malnutrition, and smoking (35). But in this study, anxiety in the multi-ethnic region of western China was not significantly associated with the different sarcopenia groups, while depression showed significant associations with severe sarcopenia.

Depression, characterized by low mood, slow thinking, disrupted sleep or appetite, and feelings of fatigue, is a common mental disorder in older adults. Some studies have found a relationship between depression and body composition, involving factors such as skeletal muscle mass, strength, and muscle function, all of which are directly related to sarcopenia (40–42). Both sarcopenia and depression are associated with reduced physical activity, upregulation of inflammatory factors, and hormonal dysregulation of the hypothalamic–pituitary–adrenal axis (43). However, no significant association has been found between sarcopenia and depression (44), while there are also reports suggesting a significant association between the two (45). Emerging evidence suggests that poor dietary quality, characterized by low protein and micronutrient intake, may exacerbate sarcopenia progression and comorbid anxiety/depression (46). According to the diagnostic criteria of 2019 AWGS for sarcopenia, we subdivided sarcopenia into different groups and collected data on depression, which further confirmed a significant correlation between depression and both diagnosed and severe sarcopenia.

However, this study also has certain limitations. Although the Pittsburgh Sleep Quality Index is a validated and reliable measure, it cannot perfectly capture sleep parameters compared to the gold standard of polysomnography. Additionally, for participants with anxiety and depression, further discussion is needed to determine if their medication status may have influenced their muscle function.

## Conclusion

This study shows that in multi-ethnic populations in western China, there is a significant association between sleep and severe sarcopenia, depression, and both diagnosed and severe sarcopenia, but no significant correlation between anxiety and sarcopenia after stratification. Preventing or treating sleep disorders and depression in the population may be effective in delaying or reducing the onset of sarcopenia, and can help formulate specific medical policies. Further longitudinal studies are needed to confirm the relationships between sleep, anxiety, depression, and sarcopenia.

## Data Availability

The raw data supporting the conclusions of this article will be made available by the authors, without undue reservation.

## References

[1] Fang EF Scheibye-Knudsen M Jahn HJ Li J Ling L Guo H. A research agenda for aging in China in the 21st century. Ageing Res Rev. (2015) 24:197–205. doi: 10.1016/j.arr.2015.08.003, PMID: 26304837 PMC5179143

[2] Marck A Antero J Berthelot G Johnson S Sedeaud A Leroy A. Age-related upper limits in physical performances. J Gerontol A Biol Sci Med Sci. (2019) 74:591–9. doi: 10.1093/gerona/gly165, PMID: 30184188

[3] Kok RM Reynolds CF 3rd. Management of depression in older adults: a review. JAMA. (2017) 317:2114–22. doi: 10.1001/jama.2017.5706, PMID: 28535241

[4] Karlsson B Johnell K Sigström R Sjöberg L Fratiglioni L. Depression and depression treatment in a population-based study of individuals over 60 years old without dementia. Am J Geriatr Psychiatry. (2016) 24:615–23. doi: 10.1016/j.jagp.2016.03.009, PMID: 27297634

[5] Malinowska KB Ikezoe T Ichihashi N Arai H Murase K Chin K. Self-reported quality of sleep is associated with physical strength among community-dwelling young-old adults. Geriatr Gerontol Int. (2017) 17:1808–13. doi: 10.1111/ggi.12965, PMID: 28060455

[6] Hofmann M Halper B Oesen S Franzke B Stuparits P Tschan H. Serum concentrations of insulin-like growth factor-1, members of the TGF-beta superfamily and follistatin do not reflect different stages of dynapenia and sarcopenia in elderly women. Exp Gerontol. (2015) 64:35–45. doi: 10.1016/j.exger.2015.02.008, PMID: 25681638

[7] Kris-Etherton PM Petersen KS Hibbeln JR Hurley D Kolick V Peoples S. Nutrition and behavioral health disorders: depression and anxiety. Nutr Rev. (2021) 79:247–60. doi: 10.1093/nutrit/nuaa025, PMID: 32447382 PMC8453603

[8] Cawthon PM Manini T Patel SM Newman A Travison T Kiel DP. Putative cut-points in sarcopenia components and incident adverse health outcomes: an SDOC analysis. J Am Geriatr Soc. (2020) 68:1429–37. doi: 10.1111/jgs.16517, PMID: 32633824 PMC7508260

[9] Xu J Wan CS Ktoris K Reijnierse EM Maier AB. Sarcopenia is associated with mortality in adults: a systematic review and meta-analysis. Gerontology. (2022) 68:361–76. doi: 10.1159/000517099, PMID: 34315158

[10] Goldman SE Stone KL Ancoli-Israel S Blackwell T Ewing SK Boudreau R. Poor sleep is associated with poorer physical performance and greater functional limitations in older women. Sleep. (2007) 30:1317–24. doi: 10.1093/sleep/30.10.1317, PMID: 17969465 PMC2266278

[11] Dam TT Ewing S Ancoli-Israel S Ensrud K Redline S Stone K. Association between sleep and physical function in older men: the osteoporotic fractures in men sleep study. J Am Geriatr Soc. (2008) 56:1665–73. doi: 10.1111/j.1532-5415.2008.01846.x, PMID: 18759758 PMC2631084

[12] Denison HJ Jameson KA Sayer AA Patel HP Edwards MH Arora T. Poor sleep quality and physical performance in older adults. Sleep Health. (2021) 7:205–11. doi: 10.1016/j.sleh.2020.10.002, PMID: 33223446

[13] Wolitzky-Taylor KB Castriotta N Lenze EJ Stanley MA Craske MG. Anxiety disorders in older adults: a comprehensive review. Depress Anxiety. (2010) 27:190–211. doi: 10.1002/da.20653, PMID: 20099273

[14] Liu X Hou L Xia X Liu Y Zuo Z Zhang Y. Prevalence of sarcopenia in multi ethics adults and the association with cognitive impairment: findings from West-China health and aging trend study. BMC Geriatr. (2020) 20:63. doi: 10.1186/s12877-020-1468-5, PMID: 32066390 PMC7027212

[15] Chen LK Woo J Assantachai P Auyeung TW Chou MY Iijima K. Asian working Group for Sarcopenia: 2019 consensus update on sarcopenia diagnosis and treatment. J Am Med Dir Assoc. (2020) 21:300–307.e2. doi: 10.1016/j.jamda.2019.12.012, PMID: 32033882

[16] Hou L Liu X Zhang Y Zhao W Xia X Chen X. Cohort profile: West China health and aging trend (WCHAT). J Nutr Health Aging. (2021) 25:302–10. doi: 10.1007/s12603-020-1530-1, PMID: 33575720 PMC12280628

[17] Wang H Hai S Cao L Zhou J Liu P Dong BR. Estimation of prevalence of sarcopenia by using a new bioelectrical impedance analysis in Chinese community-dwelling elderly people. BMC Geriatr. (2016) 16:216. doi: 10.1186/s12877-016-0386-z, PMID: 28031025 PMC5198494

[18] Tosato M Marzetti E Cesari M Savera G Miller RR Bernabei R. Measurement of muscle mass in sarcopenia: from imaging to biochemical markers. Aging Clin Exp Res. (2017) 29:19–27. doi: 10.1007/s40520-016-0717-0, PMID: 28176249

[19] Toussaint A Hüsing P Gumz A Wingenfeld K Härter M Schramm E. Sensitivity to change and minimal clinically important difference of the 7-item generalized anxiety disorder questionnaire (GAD-7). J Affect Disord. (2020) 265:395–401. doi: 10.1016/j.jad.2020.01.032, PMID: 32090765

[20] Shin C Park MH Lee SH Ko YH Kim YK Han KM. Usefulness of the 15-item geriatric depression scale (GDS-15) for classifying minor and major depressive disorders among community-dwelling elders. J Affect Disord. (2019) 259:370–5. doi: 10.1016/j.jad.2019.08.053, PMID: 31470180

[21] von Haehling S Morley JE Anker SD. An overview of sarcopenia: facts and numbers on prevalence and clinical impact. J Cachexia Sarcopenia Muscle. (2010) 1:129–33. doi: 10.1007/s13539-010-0014-2, PMID: 21475695 PMC3060646

[22] Lin CC Lin WY Meng NH Li CI Liu CS Lin CH. Sarcopenia prevalence and associated factors in an elderly Taiwanese metropolitan population. J Am Geriatr Soc. (2013) 61:459–62. doi: 10.1111/jgs.12129, PMID: 23496184

[23] Cole CS Richards KC Beck CC Roberson PK Lambert C Furnish A. Relationships among disordered sleep and cognitive and functional status in nursing home residents. Res Gerontol Nurs. (2009) 2:183–91. doi: 10.3928/19404921-20090527-01, PMID: 20078008 PMC2861436

[24] Morgan K Hartescu I. Sleep duration and all-cause mortality: links to physical activity and prefrailty in a 27-year follow up of older adults in the UK. Sleep Med. (2019) 54:231–7. doi: 10.1016/j.sleep.2018.11.008, PMID: 30584984

[25] Barrea L Frias-Toral E Aprano S Castellucci B Pugliese G Rodriguez-Veintimilla D. The clock diet: a practical nutritional guide to manage obesity through chrononutrition. Minerva Med. (2022) 113:172–88. doi: 10.23736/S0026-4806.21.07207-4, PMID: 33913659

[26] Prokopidis K Dionyssiotis Y. Effects of sleep deprivation on sarcopenia and obesity: a narrative review of randomized controlled and crossover trials. J Frailty Sarcopenia Falls. (2021) 6:50–6. doi: 10.22540/JFSF-06-050 34131601 PMC8173530

[27] Cholewa JM Dardevet D Lima-Soares F de Araújo Pessôa K Oliveira PH Dos Santos Pinho JR. Dietary proteins and amino acids in the control of the muscle mass during immobilization and aging: role of the MPS response. Amino Acids. (2017) 49:811–20. doi: 10.1007/s00726-017-2390-9, PMID: 28175999

[28] Kwon YJ Jang SY Park EC Cho AR Shim JY Linton JA. Long sleep duration is associated with sarcopenia in Korean adults based on data from the 2008-2011 KNHANES. J Clin Sleep Med. (2017) 13:1097–104. doi: 10.5664/jcsm.6732 28760192 PMC5566466

[29] de Sá Souza H de Melo CM Piovezan RD Miranda REEPC Carneiro-Junior MA Silva BM. Resistance training improves sleep and anti-inflammatory parameters in sarcopenic older adults: a randomized controlled trial. Int J Environ Res Public Health. (2022) 19:16322. doi: 10.3390/ijerph192316322 36498393 PMC9736460

[30] Vitale JA Bonato M La Torre A Banfi G. The role of the molecular clock in promoting skeletal muscle growth and protecting against sarcopenia. Int J Mol Sci. (2019) 20:4318. doi: 10.3390/ijms20174318, PMID: 31484440 PMC6747101

[31] Lipton JO Yuan ED Boyle LM Ebrahimi-Fakhari D Kwiatkowski E Nathan A. The circadian protein BMAL1 regulates translation in response to S6K1-mediated phosphorylation. Cell. (2015) 161:1138–51. doi: 10.1016/j.cell.2015.04.002, PMID: 25981667 PMC4447213

[32] Shibuki T Iida M Harada S Kato S Kuwabara K Hirata A. The association between sleep parameters and sarcopenia in Japanese community-dwelling older adults. Arch Gerontol Geriatr. (2023) 109:104948. doi: 10.1016/j.archger.2023.104948, PMID: 36764202

[33] Luik AI Zuurbier LA Direk N Hofman A Van Someren EJ Tiemeier H. 24-hour activity rhythm and sleep disturbances in depression and anxiety: a population-based study of middle-aged and older persons. Depress Anxiety. (2015) 32:684–92. doi: 10.1002/da.22355, PMID: 25693731

[34] Dittoni S Mazza M Losurdo A Testani E di Giacopo R Marano G. Psychological functioning measures in patients with primary insomnia and sleep state misperception. Acta Neurol Scand. (2013) 128:54–60. doi: 10.1111/ane.12078, PMID: 23406317

[35] Difrancesco S Lamers F Riese H Merikangas KR Beekman ATF Hemert AM. Sleep, circadian rhythm, and physical activity patterns in depressive and anxiety disorders: a 2-week ambulatory assessment study. Depress Anxiety. (2019) 36:975–86. doi: 10.1002/da.22949, PMID: 31348850 PMC6790673

[36] Castrén E Kojima M. Brain-derived neurotrophic factor in mood disorders and antidepressant treatments. Neurobiol Dis. (2017) 97:119–26. doi: 10.1016/j.nbd.2016.07.010, PMID: 27425886

[37] Dalle S Rossmeislova L Koppo K. The role of inflammation in age-related sarcopenia. Front Physiol. (2017) 8:1045. doi: 10.3389/fphys.2017.01045, PMID: 29311975 PMC5733049

[38] Chen L Liang J Wen J Huang H Li L Lin W. Is waist circumference a negative predictor of calcaneal bone mineral density in adult Chinese men with normal weight? Ann Transl Med. (2019) 7:201. doi: 10.21037/atm.2019.04.71, PMID: 31205919 PMC6545315

[39] Moylan S Eyre HA Maes M Baune BT Jacka FN Berk M. Exercising the worry away: how inflammation, oxidative and nitrogen stress mediates the beneficial effect of physical activity on anxiety disorder symptoms and behaviours. Neurosci Biobehav Rev. (2013) 37:573–84. doi: 10.1016/j.neubiorev.2013.02.003, PMID: 23415701

[40] Gariballa S Alessa A. Associations between low muscle mass, blood-borne nutritional status and mental health in older patients. BMC Nutr. (2020) 6:6. doi: 10.1186/s40795-019-0330-7, PMID: 32190345 PMC7066831

[41] Yuenyongchaiwat K Buranapuntalug S Pongpanit K Kulchanarat C Satdhabudha O. Anxiety and depression symptomatology related to inspiratory muscle strength and functional capacity in preoperative cardiac surgery patients: a preliminary cross-sectional study. Indian J Psychol Med. (2020) 42:549–54. doi: 10.1177/0253717620930318 33354081 PMC7735231

[42] Moon JH Kong MH Kim HJ. Low muscle mass and depressed mood in Korean adolescents: a cross-sectional analysis of the fourth and fifth Korea National Health and nutrition examination surveys. J Korean Med Sci. (2018) 33:e320. doi: 10.3346/jkms.2018.33.e320, PMID: 30534032 PMC6281954

[43] Hallgren M Herring MP Owen N Dunstan D Ekblom Ö Helgadottir B. Exercise, physical activity, and sedentary behavior in the treatment of depression: broadening the scientific perspectives and clinical opportunities. Front Psych. (2016) 7:36. doi: 10.3389/fpsyt.2016.00036 PMC4786540 27014101

[44] Patino-Hernandez D David-Pardo DG Borda MG Pérez-Zepeda MU Cano-Gutiérrez C. Association of Fatigue with Sarcopenia and its elements: a secondary analysis of SABE-Bogotá. Gerontol Geriatr Med. (2017) 3:2333721417703734. doi: 10.1177/2333721417703734, PMID: 28474000 PMC5407660

[45] Chang KV Hsu TH Wu WT Huang KC Han DS. Is sarcopenia associated with depression? A systematic review and meta-analysis of observational studies. Age Ageing. (2017) 46:738–46. doi: 10.1093/ageing/afx094, PMID: 28633395

[46] Romano F Muscogiuri G Di Benedetto E Zhukouskaya VV Barrea L Savastano S. Vitamin D and sleep regulation: is there a role for vitamin D? Curr Pharm Des. (2020) 26:2492–6. doi: 10.2174/1381612826666200310145935, PMID: 32156230

