# Supplementary material for: Associations between sleep disorders, anxiety, depression, and the phases of sarcopenia to severe sarcopenia: findings from the WCHAT study
Source: Front Public Health. 2025 Aug 28;13:1539729. doi: 10.3389/fpubh.2025.1539729 (PMC12424590; doi:10.3389/fpubh.2025.1539729)

Supplementary figures. The distribution of GAD-7 scores, GDS-15 scores, PSQI scores and SMI in different sarcopenia groups.


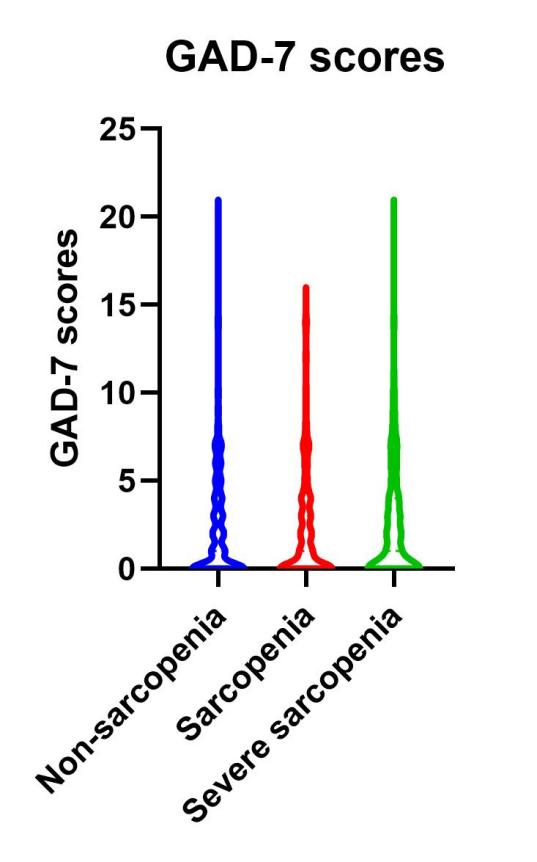

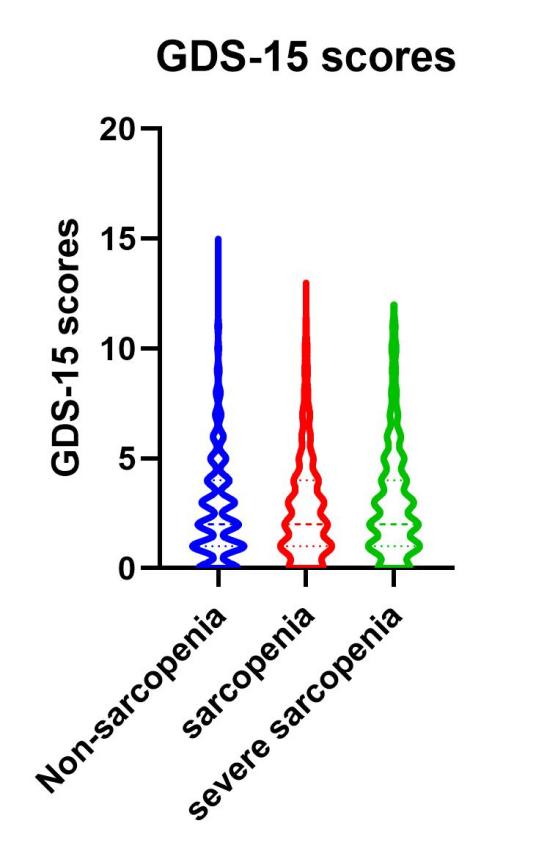


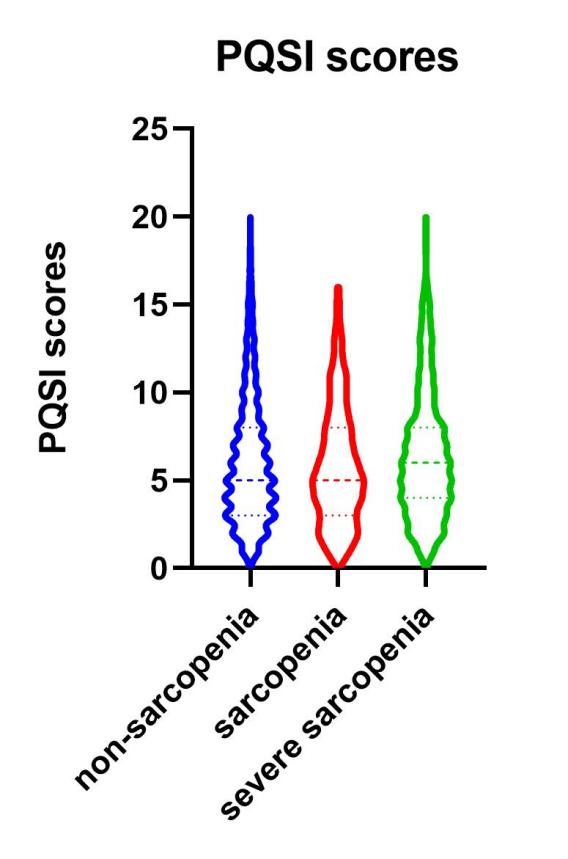

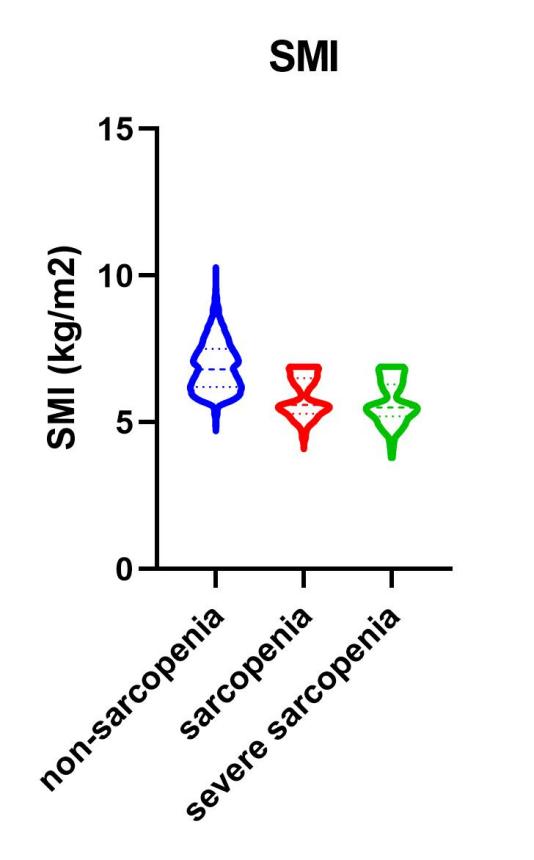

Supplement: Supplementary file 1 [file Supplementary_file_1.docx]
